# Risks of Adverse Neonatal Outcomes in Early Adolescent Pregnancy Using Group Prenatal Care as a Strategy for Public Health Policies: A Retrospective Cohort Study in Brazil

**DOI:** 10.3389/fpubh.2021.536342

**Published:** 2021-04-09

**Authors:** Danylo José Palma Honorato, Izabela Fulone, Marcus Tolentino Silva, Luciane Cruz Lopes

**Affiliations:** Pharmaceutical Sciences Graduate Course, University of Sorocaba (UNISO), Sorocaba, Brazil

**Keywords:** pregnant, prematurity, low birth weight, prenatal care, adolescents

## Abstract

**Background:** Adolescent pregnancy is a public health concern and many studies have evaluated neonatal outcomes, but few have compared younger adolescents with older using adequate prenatal care.

**Objective:** To compare the risks of adverse neonatal outcomes in younger pregnant adolescents who are properly followed through group prenatal care (GPC) delivered by specialized public services.

**Methods:** This retrospective cohort study followed pregnant adolescents (aged 10–17 years) who received GPC from specialized public services in Brazil from 2009 to 2014. Data were obtained from medical records and through interviews with a multidisciplinary team that treated the patients. The neonatal outcomes (low birth weight, prematurity, Apgar scores with 1 and 5 min, and neonatal death) of newborns of adolescents aged 10–13 years were compared to those of adolescents aged 14–15 years and 16–17 years. Incidence was calculated with 95% confidence intervals (CIs) and compared over time using a chi-squared test to observe trends. Poisson Multivariate logistic regression was used to adjust for confounding variables. The results are presented as adjusted relative risks or adjusted mean differences.

**Results:** Of the 1,112 adolescents who were monitored, 758 were included in this study. The overall incidence of adverse neonatal outcomes (low birth weight and prematurity) was measured as 10.2% (95% CI: 9.7–11.5). Apgar scores collected at 1 and 5 min were found to be normal, and no instance of fetal death occurred. The incidence of low birth weight was 16.1% for the 10–13 age group, 8.7% for the 14–15 age group and 12.1% for the 16–17 age group. The incidence of preterm was measured at 12, 8.5, and 12.6% for adolescents who were 10–13, 14–15, and 16–17 years of age, respectively. Neither low birth weight nor prematurity levels significantly differed among the groups (*p* > 0.05). The infants born to mothers aged 10–13 years presented significantly (*p* < 0.05) lower Apgar scores than other age groups, but the scores were within the normal range.

**Conclusions:** Our findings showed lower incidence of neonatal adverse outcomes and no risk difference of neonatal outcomes in younger pregnancy adolescents. It potentially suggests that GPC model to care pregnant adolescents is more important than the age of pregnant adolescent, however further research is needed.

## Introduction

Adolescent pregnancy is a public health concern due to the complexities and impacts of problems related to it (1). Among adolescents, pregnancy is seldom planned or desired; in many cases, it results from abuse or sexual violence, causing permanent changes in their lives and creating a cycle of inequality, social exclusion, difficulty attending school, job loss, or reductions in work hours and the potential development of emotional disorders (2). The adolescent's life course changes, eventually leading to school dropouts and the perpetuation of poverty, inequality, and exclusion (3).

Approximately 12 million adolescents aged 15–19 years and 770 thousand of whom are under 15 years give birth each year in developing world (4). In Brazil, the rate of adolescent pregnancy is 400 thousand cases each year (5).

Adolescent pregnancy may be a risk factor for adverse obstetric, maternal, or neonatal outcomes such as prematurity, low birth weight, low Apgar scores, and neonatal and maternal mortality, mainly in mothers under 15 years of age (6, 7). One study reported an increase in maternal and fetal complications occurring during all stages of pregnancy among pregnant adolescents (8, 9). However, according to other research, such complications are more closely associated with newborns, particularly for cases of prematurity, low birth weight and death (8, 10, 11).

Factors potentially associated with adverse neonatal outcomes include a reduced number of prenatal visits being made, late or inadequate access to prenatal care, race, marital status, low education level, smoking and poverty (12, 13).

Preventive interventions to address preterm low birth weight and mortality include efforts to improve the quality of prenatal care in the intrapartum and postpartum periods (14). Although Brazil maintains elevated levels of prenatal care coverage across the country, quality, and adequacy rates are low, ranging from 4.5 to 66.1% in several regions (12). This is attributed to individuals not making the ideal number of consultations or using early assistance services, to failures of basic procedures and to a lack of content designed for consultations (15).

Recently, group prenatal care models in general, both for adult women with risk factors and for adolescents, have been growing in popularity internationally, mainly in high-income settings; such models may offer advantages over the traditional individual model of care (16). Complete prenatal care is provided in a group setting and integrates pregnancy health assessments and education on nutrition; potential problems with pregnancy or childbirth; child care; the prevention or detection of disease; skills building services; and peer support (14). This model has been adopted in Brazil and allows for significantly more time compared to models of traditional individual prenatal care, provides pregnancy care to ~12 women simultaneously over up to 10, 90–120-min group visits and follows American Congress of Obstetricians and Gynecologists guidelines (17, 18).

A recent overview shows that compared to findings associated with individual care, group prenatal care is associated with a decreased rate of low birth weight overall and with a reduction in risk for preterm birth among African-American women (16). A systematic review (with only two high quality randomized studies conducted on women with high-risk pregnancies) suggests that compared to standard individual care, group prenatal care decreases rates of preterm births and cesarean sections and increases the prevalence of breastfeeding and satisfaction with care (19). These improved outcomes may be due to reduced levels of stress and enhanced knowledge, and stronger effects have been observed with women who experience higher levels of stress (20).

The data are conflicting, and evidence of benefits in low- and middle-income countries (where resources are scarce) is limited (21). More researches are needed to demonstrate the health impact and effects of group prenatal care in these settings.

Considering the high prevalence of adolescent pregnancy and its consequences in Brazil, the aim of the present study was to compare the risks of adverse neonatal outcomes in younger pregnant adolescents who are properly followed through group prenatal care delivered by specialized public services.

## Methods

### Design and Setting

This is a retrospective cohort study of pregnant adolescents cared for in the public health system in Sorocaba city, State of São Paulo, Brazil.

The study was conducted at the Edward Maluf Municipal Polyclinic (EMMP), Sorocaba, using medical records for pregnant women who received prenatal care from 2009 to 2014. As a specialized service, the Municipal Polyclinic is a reference center for adolescent prenatal care located in the city of Sorocaba. Sorocaba is located in the state of São Paulo, and according to the last census performed by the Brazilian Institute of Geography and Statistics (IBGE), its estimated population in 2013 was 629,231 inhabitants (22).

In 2000, after some changes to women's health policies were made, the Brazilian Health Ministry launched the Prenatal and Birth Humanization Program to improve the coverage and quality of and access to prenatal, delivery and postpartum care for women and newborns (23). The Prenatal and Birth Humanization Program follows a protocol involving several tests, follow-up routines, recording of prenatal visits, laboratory and imaging tests, and registration of all pregnant women during their respective visits using specific software. Furthermore, the program follows recommendations from the American Congress of Obstetricians and Gynecologists on group prenatal care (17, 18).

Public health services offered by the EMMP for pregnant adolescents (23) apply principles and best features of the group prenatal care model, including three main elements: (i) health assessment, (ii) education and skills building (focusing on nutrition, exercise, pregnancy issues, infant care and feeding, pregnancy comfort measures, childbirth preparation, etc.) and (iii) peer support (15, 24, 25). Generally, the groups meet eight to 10 times during the pregnancy period, and care is provided to groups of 8–12women through 120-min sessions.

The service is provided by a multidisciplinary team that includes obstetricians, obstetric nurses, psychologists, social workers, and dentists. In addition, as the polyclinic is a specialized healthcare service, referrals to other medical specialists and for emergency obstetric ultrasounds are easily available. To promote adherence to prenatal care, various social activities are offered to pregnant women to promote their social and occupational inclusion. For women who live far away and have difficulty attending visits, transportation assistance is provided through municipal buses. Whenever a woman misses an appointment, she receives a phone call on the day of the appointment to identify her reason for missing the appointment and to schedule a new visit.

### Participants

As explained in the previous item, the EMMP is referenced by all outpatient clinics who refer pregnant women at risk and adolescents for this follow-up. Thus, all pregnant adolescents cared by the public health system in Sorocaba and the region performed their prenatal care at the EMMP. This study included all adolescents who use public health services, at any stage of pregnancy under 18 years of age and who received care at the EMMP. From this, we excluded miscarriage cases or records with missing data.

Among adolescents, pregnancy is classified in girls of up to 15 years of age and in girls older than 16 years of age. Pregnancy adolescents up to 15 years are considered to be at a high risk of adverse maternal, obstetric, and neonatal outcomes (26). In the present study, the adolescents were intentionally divided into three age groups to achieve a stronger understanding of differences observed within narrower age ranges. We were also interested in analyzing the youngest participants (those younger than 13 years of age) to address a lack of data in the literature on this population.

### Variables and Data Source

All of the adolescents included in the study were followed up until the end of the pregnancy period. The participants were divided by age as follows: 10–13, 14–15, and 16–17 years of age.

The extraction of variables was done using an electronic form developed specifically for this study. The investigator DPH, filled out each electronic form extracting information directed from the medical record and cross-checked with the information in the database of the EMMP. When necessary, the multidisciplinary teams were interviewed to address any questions about the medical records.

General and socio-demographic characteristics (enrolment date, birth date, employment status of the head of the family, having a partner, literacy level, having another child under 2 years of age, receiving child benefits and whether the pregnancy was desired) were collected from the polyclinic's follow-up service enrolment form at the time of prenatal care enrolment.

The following information was collected from the participants' medical records: (i) prenatal and pregnancy-related characteristics (referring ambulatory unit, prenatal care start date, gestational weeks at the start of prenatal care, number of prenatal visits, last menstrual period, expected delivery date and infant's gestational age at delivery); (ii) birth-related characteristics (delivery date, place and type; preterm or full-term birth; Apgar score at 1 and 5 min; and neonate's birth weight); (iii) clinical characteristics (presence of comorbidities such as human immunodeficiency virus (HIV) or venereal disease and findings on serology tests); and (iv) obstetric characteristics (vaginal birth, cesarean section and forceps assisted).

Risky neonatal outcomes were defined as follows: low birth weight: <2,500 g; prematurity: birth that occurs after the 22nd week of gestation and before the 37th; mean Apgar score at 1 and 5 min; and neonatal death.

### Statistical Analysis

The variables of interest were summarized as descriptive statistics; frequency was calculated for categorical variables, and the mean and standard deviation were calculated for continuous variables.

The differences in demographic, clinical, and obstetric characteristics, low weight, and prematurity between age groups were calculated using the chi-square test or fisher's exact test (expected values <5). Differences in Apgar scores at 1 and 5 min were calculated by ANOVA.

The association between maternal age and Apgar score was determined by calculating coefficients generated from a multiple regression model adjusted for covariables infant gender, maternal comorbidities, delivery type, positive HIV status and positive VDRL.

The association between maternal age and the outcomes of low birth weight and preterm birth was estimated by Poisson regression (27). For this purpose, the incidences of low weight and preterm between the groups were calculated and proceeded with adjustments with the variables of the newborn's sex and mother's comorbidities.

To minimize chance effects, sensitivity analyses were performed by repeating the same calculations on random subsamples (bootstrapping) (27). Bonferroni correction was used to conservatively estimate the statistically significant variables. Imputation was not performed for missing data; in such cases, the corresponding information was excluded from the multivariate analysis. All analyses were performed in STATA (version 14) with a 95% confidence interval (95% CI); the significance level (alpha error) was set to 5%.

### Ethical Approval

This study was approved by the Research Ethics Committee of the University of Sorocaba, which is accredited by the National Research Ethics Commission according to stipulations of the National Health Council Ordinance 466/2012; it was also approved according to Platform Brazil Protocol no. 985.590 for March 13, 2015. EMMP also approved the protocol and personal information of the participants was kept blinded to investigators. Waiver of parental permission (Written informed consent from the participants' legal guardian) was requested and authorized by Research Ethics Committee.

## Results

**Figure 1:**
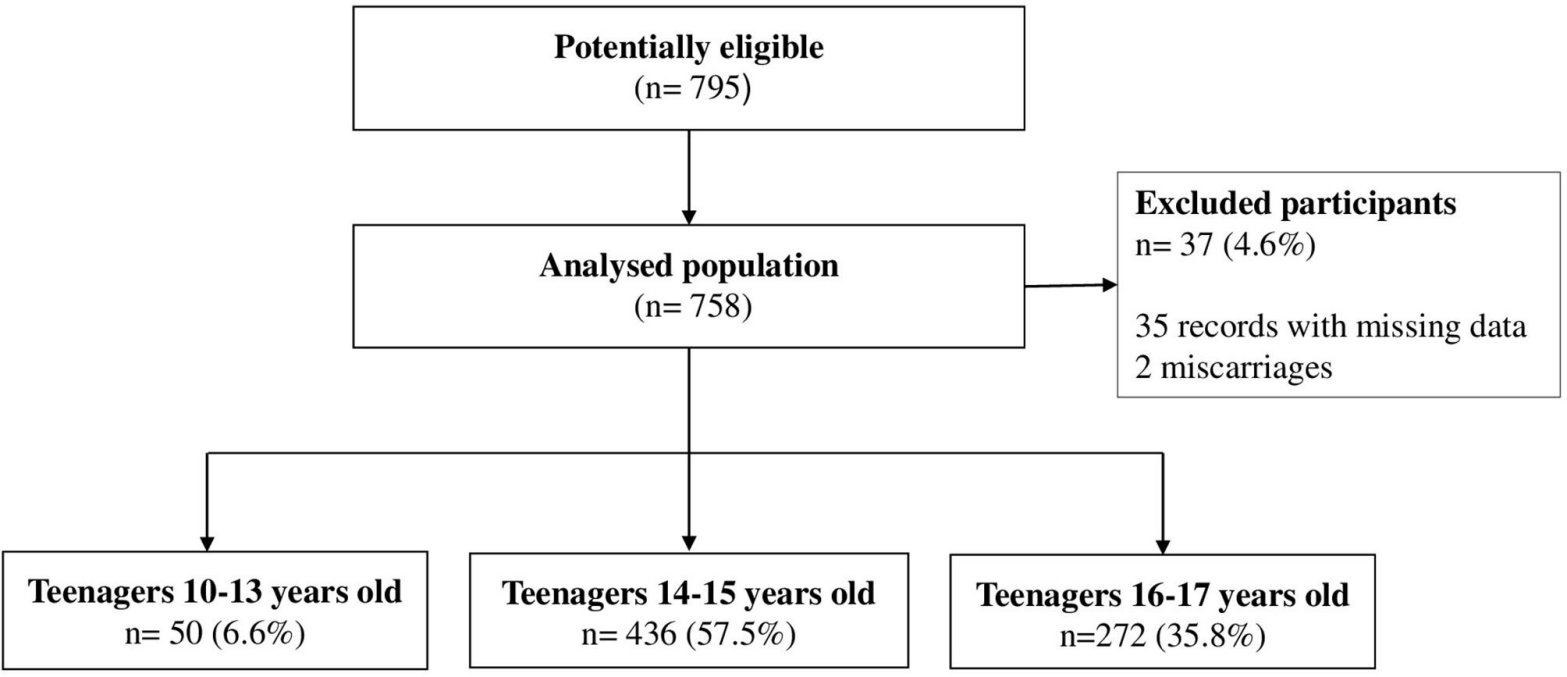
Flowchart representing the sample composition.

During the 5-year study period, 795 adolescents enrolled in the Sorocaba municipal government's high-risk prenatal care program were identified, and 758 were included in the final sample. A total of 37 (4.6%) pregnant adolescents were excluded because they had miscarried, or because their medical records had missing data. None of the studied adolescents (*n* = 758) were lost to analysis (Figure 1).

Table 1 describes socio-demographic, clinical and obstetric characteristics of the sample. Approximately 79.8% of the participants started prenatal care during the first trimester of pregnancy, and 96% made six visits or more. Vaginal birth was the most frequent delivery type across all of the groups (68.6%). There was a difference in the distribution between the hospitals (*p* = 0.003) and the presence of comorbidities (*p* < 0.001) between the age groups. There was no statistical difference between lower preterm birth rates and the hospitals: hospital A (*p* = 0.383); hospital B (*p* = 1.00); hospital C (*p* = 0.406).

**Table 1:** Socio-demographic, clinical, and obstetric characteristics of pregnant adolescents who received prenatal care services.

**Variables**	**Total**	**Age 16–17**	**Age 14–15**	**Age 10–13**	***p-*value**
	***n* (%)**	***n* (%)**	***n* (%)**	***n* (%)**	
	758 (100)	272 (35.8)	436 (57.5)	50 (6.6)	
**SOCIO-DEMOGRAPHIC CHARACTERISTICS**					
***Unemployed head of family***					
Yes	24 (3.2)	9 (3.3)	13 (3.0)	2 (4.0)	0.793
No	725 (96.8)	261 (96.7)	416 (97.0)	48 (96.0)	–
***Has child < 2 years of age***					
Yes	21 (2.8)	8 (3.0)	12 (2.8)	1 (2.0)	1.000
No	733 (97.2)	261 (97.0)	423 (97.2)	49 (98.0)	–
***Partner***					
Yes	555 (73.2)	205 (75.4)	315 (72.2)	35 (70.0)	0.557
No	203 (26.8)	67 (24.3)	121 (27.8)	15 (30.0)	–
***Self-reported literacy***					
Yes	749 (99.1)	268 (98.9)	431 (99.1)	50 (100)	1.000
No	7 (0.9)	3 (1.1)	4 (0.9)	0.00	–
***Desired infant***					
Yes	754 (99.6)	270 (99.6)	434 (99.5)	50 (100)	1.000
No	3 (0.4)	1 (0.4)	2 (0.5)	0.00	–
***Prenatal care start***					
First trimester	554 (79.8)	208 (81.2)	308 (78)	38 (88.4)	0.369
Second trimester	121(17.5)	39 (15.2)	77 (19.5)	5 (11.6)	–
Third trimester	19 (2.7)	9 (3.5)	10 (2.5)	0.00	–
***Number of prenatal visits***					
< six visits	30 (4.01)	9 (3.4)	16 (3.7)	5 (10.0)	0.106
Six or more visits	718 (96.0)	257 (96.6)	416 (96.3)	45 (90.0)	
***Delivery hospital***					
Hospital A	435 (59.2)	158 (62.1)	239 (55.5)	38 (77.6)	0.003
Hospital B	176 (23.9)	47 (18.3)	121 (28.0)	8 (16.3)	–
Hospital C	124 (16.9)	50 (19.6)	71 (16.5)	3 (6.1)	–
**CLINICAL CHARACTERISTICS**					
Comorbidities	131 (17.3)	89 (32.7)	39 (8.9)	3 (6.0)	<0.001
VDRL positive	9 (1.2)	4 (1.5)	5 (1.2)	0.00	0.859
HIV positive	2 (0.2)	1 (0.4)	1 (0.2)	0.00	1.000
**OBSTETRIC CHARACTERISTICS**					
Vaginal birth	504 (68.6)	169 (66.2)	302 (70.1)	33 (67.4)	0.081
Cesarean section	203 (27.6)	81 (31.8)	110 (25.5)	12 (24.5)	–
Forceps assisted	28 (3.8)	5 (2.0)	19 (4.4)	4 (8.1)	–

Prematurity occurred in 10.2% of the newborns, a proportion similar to that found for low birth weight (10.4%). The incidence of low birth weight was 16.1% for the 10–13 age group, 8.7% for the 14–15 age group and 12.1% for the 16–17 age group. The incidence preterm risk was measured as 12, 8.5, and 12.6% for adolescents that were 10–13, 14–15, and 16–17 years of age, respectively.

The Apgar scores at 1 and 5 min were found to be slightly lower for the infants of mothers aged 10–13 years (Table 2).

**Table 2:** Characteristics of infants born to mothers who received prenatal care.

**Variables**	**Total**	**Ages 16–17**	**Ages 14–15**	**Ages 10–13**	***P*-value**
	*n* = 758 (100%)	*n* = 272 (35.8%)	*n* = 436 (57.5%)	*n* = 50 (6.6%)	
**Gender**					
Male	388 (52.8)	129 (50.6)	236 (54.8)	23 (46.9)	0.401
Female	347 (47.2)	126 (49.4)	195 (45.2)	26 (53.1)	
**Apgar (mean** **±** **standard deviation)**					
Apgar at 1 min	7.83 ± 1.62	7.93 ± 1.50	7.84 ± 1.65	7.28 ± 1.96	0.035
Apgar at 5 min	8.99 ± 0.72	9.00 ± 0.73	9.01 ± 0.69	8.76 ± 0.94	0.056
**Birth weight**					
Low weight	79 (10.4)	33 (12.1)	38 (8.7)	8 (16.1)	0.132
**Prematurity**					
Term	676 (89.8)	236 (87.4)	396 (91.4)	44 (88)	0.197
Preterm	77 (10.2)	34 (12.6)	37 (8.5)	6 (12.0)	

Table 3 describes the outcomes of infants born to the adolescent mothers. Neither low birth weight nor prematurity levels significantly differed among the groups (*p* > 0.05). Vitality by Apgar score is lower in ages 10–13 group than ages 16–17 group. Ages 14–15 group did not show differences between ages 16–17 group.

**Table 3:** Mean difference and relative risk of neonatal outcomes.

**Outcome**	Ages 16–17^**^	Ages 14–15^*^	***P-*value**	Ages 10–13^*^	***P-*value**
	*n* = 272	*n* = 436		*n* = 50	
**Apgar coeff (95% CI)**^**&**^					
At 1 min	Ref^**^	– 0.10 (−0.36 to 0.15)	0.43	−0.69 (−1.19 to −0.19)	0.007
At 5 min	Ref^**^	0.03 (−0.09 to 0.14)	0.63	−0.26 (−0.49 to −0.04)	0.021
**Birth weight RR (95% CI)**					
Low weight	1.00	0.76 (0.47 to 1.23)	0.26	1.48 (0.68 to 3.23)	0.320
**Prematurity RR (95% CI)**					
Preterm	1.00	0.72 (0.44 to 1.16)	0.17	1.05 (0.45 to 2.60)	0.860

& *Mean difference; RR, relative risk; IC, confidence interval*.

* Analysis adjusted to infant gender, maternal comorbidities, delivery type, HIV-positive status, and VDRL positive status;

** *Reference group for multivariate analysis*.

The infants born to mothers aged 10–13 years presented significantly lower Apgar scores, with a mean reduction of 0.69 points at 1 min and 0.26 points at 5 min. These results remained significant through the sensitivity analysis. No instance of neonatal death occurred.

## Discussion

### Main Findings

The results of the study showed that pregnant adolescents, disaggregated across three thresholds of age at pregnancy, did not exhibit difference between the adverse neonatal outcomes. The overall incidences of low birth weight and prematurity were 10.4 and 10.2%, respectively. Vitality by Apgar scores at 1 and 5 min were normal (values of Apgar > 7) in all groups, although slightly lower for the infants of mothers aged 10–13 years. No fetal death occurred during the study period, and vaginal birth was the most frequent delivery type across all of the groups.

### Relationship to Previous Studies

According to World Health Organization (WHO), prenatal care should start early, be universally available, be performed periodically and be integrated with all other preventive and therapeutic actions (28). Also, prenatal care should be adapted for adolescents in order to provide them easy access to multidisciplinary care to compensate for a lack of or delay in care (29). The success of prenatal care largely depends on its time of onset and on the number of prenatal visits made. Adolescents often have low adherence to prenatal care and fewer prenatal visits, especially in the first trimester (29, 30). In the present study, we could assume higher adherence to the prenatal care compared with the national average due to high rate (82%) of time of onset in first semester and number of prenatal visits made (more than six) that we could assume higher adherence to the prenatal care compared with the national average.

Although not have compared to individualized care, we could suggest that the good engagement of these adolescents to this program is due the quality of prenatal care provided in primary care and the connection with the specialized services in this region, which comply with all recommendations established by the Brazilian Health Ministry and WHO (23, 28). In addition to routine tests, vaccinations, multidisciplinary care and social and educational activities, all of which are duly recorded in a universal open-access system, contribute to the satisfactory provision of services.

We cannot affirm with certainty that the positive outcomes obtained in this study is specifically due to the GPC model, nevertheless our results reinforce other findings (21, 31), which point out that an adequate adaptation in the implementation of the GPC model may generate robust adherence as demonstrated in this sample (>90% of the adolescents with more than six visits and ~80% starting the prenatal care in the first trimester) and contribute to better neonatal and obstetric outcomes.

Other studies revealed a high incidence and prevalence of adverse neonatal outcomes in adolescents (32, 33), mainly in pregnant with social disadvantage (34, 35). Although we have not investigated the socio-economic characteristics of these adolescents, most of the people assisted by the public health system in Brazil have lower economic conditions, but even so, they showed positive outcomes. Other researcher suggested that the prenatal care is more important than the woman's age (36), the fact that corroborates with this studied context.

Patients in group prenatal care are more satisfied and engaged in care services and adhere more to recommendations, which could have reflected in the observed outcomes. The group model based on the premise that prenatal care is more effective when learning and support are enhanced by group resources through the guidance of a professional care provider and on the notion that high-quality care cannot be achieved through traditional care services, especially for high-risk groups such as adolescents pregnant women (17).

Making the difficult transition from individual to group prenatal care takes effort and a desire to work with innovation and multidisciplinary staff. The decision for changing to introduce group prenatal care to this region could improve the quality of care. Positive experiences of the implementation of group care have also been observed in New Haven, Atlanta, New York, Boston and Iran (17, 37, 38).

Prematurity and low birthweight, are outstanding factors related to perinatal morbidity and mortality rates. Premature births are those occurring after gestational week 22 and before week 37, and in the present study, the prematurity rate (10.2%) was found to be lower than rates reported in other studies on adolescents in Brazil (39–41); the exception is one study performed in Campinas (42), in which the rate was 7.5%. None of these studies found a prematurity rate lower than that of developed countries (7%). Furthermore, no significant differences were observed in the proportion of preterm births among younger and older adolescents, in contrast to the results of other studies, in which younger adolescents were show to have increased risk of preterm delivery (43–46).

Studies have found a correlation between low birth weight and maternal age, showing that the odds of delivering a low birth-weight infant are higher for mothers under the age of 16 (47–49). However, in the present study, we found no significant difference in the rate of low birth-weight infants as a function of the maternal age ranges studied. In addition, the rate of low birth weight infants was found to be lower than that reported by another study, where in adolescents aged 11–14 years had higher risks of infants with low and very low birth weights (46, 50).

Low birth weight and prematurity is reflected in the higher Apgar scores found for most of the newborns (51, 52). The Apgar scores reporting the status of the newborns' physiologies at 1 min and at 5 min immediately after birth summarize five components: respiratory effort, color, muscle tone, heart rate, and reflex (52). The gestational age affects this score and may be reflected in lower Apgar score, which in turn may increase the relative risk of neurologic disability (7, 52). But, the Apgar scores at 1 and 5 min were normal (>7) in all groups of this study.

Younger maternal age was associated with greater risk of fetal death in some studies (45, 53), which was different to our results. No instance of fetal death occurred during the study period and this may be attributed to the resulting outcomes and especially to the low frequency of prematurity and low birth weight.

The proportion of cesarean sections was measured at 27.6% in the present study, which is higher than the level of 15% recommended by the WHO for both adult and adolescent females. The WHO has not yet established a consensus on the ideal rate of cesarean sections for the total population of women, and thus, we are still far from identifying a specific recommendation for adolescents (54).

On the other hand, given that the bodies of girls under 13 years of age are still developing, a higher frequency of cephalopelvic disproportion would be expected with a consequently higher number of cesarean sections; however, the opposite was observed. The proportion of cesarean sections was found to be considerably lower among the youngest adolescents relative to the older adolescents, corroborating the results of other studies among younger adolescents (46, 50). This finding may be explained by the larger number of forceps-assisted vaginal births involved in the study.

### Study Limitations and Strengths

The study reflects real-life data from representative sample of adolescents assisted by SUS through group prenatal care, which the results can be extrapolated to similar contexts. It is worth to mentioning that the Brazilian public health system assists ~80% of country's population. Most studies investigate teenage pregnancy without considering the quality of prenatal care services. In addition to applying well-defined eligibility criteria and controlling confounders in the analysis, this study described data collected from the real-life conditions of a public health service structured to follow up with pregnant teenagers in an adequate manner.

To ensure the internal validity of the study, we consider the following aspects. We included all pregnant adolescents assisted by the public sector in the study and only excluded those with incomplete data, which reduced the selection bias. We checked the consistency of the data contained in the medical records with the information obtained from the professionals' team and so, the memory bias was also reduced. Data were collected in the same way for all participants, avoiding performance and measurement bias. There was no misclassification of the outcomes because the adolescents were monitored from the beginning and the outcomes were not present at the beginning of the follow-up. We took care that the analyzes were made considering adjustments for confounders (sex infant, maternal comorbidities, type of delivery, HIV serology, and positive VDRL) and we performed a stratified analysis by subgroups and Poisson Multivariate logistic regression.

Although the total sample was relatively large, the subgroup analysis reduced its size, which might have influenced some of the assessed outcomes. This study also did not compare outcomes against those of adolescents not used group care or other external comparator, so it lacked a control group. We provided outcomes of internal comparisons of age groups within the adolescence age range. We also did not captured data from private sector. However, the public sector covers about 80% of the Brazilian population.

## Conclusion

The neonatal outcomes, including Apgar scores, low frequencies of prematurity, and low birth weight were within the normal range and did not differ between the age groups of pregnant adolescents who were enrolled in GPC through specialized public services which were implemented in Sorocaba.

The results suggest that adherence to the prenatal model implemented in the public health care system in Brazil could among other factors, limit the occurrence of adverse neonatal and obstetrics outcomes, even among the youngest pregnant adolescents.

In addition, we would like to suggest that future research will overcome the challenges and limitations of our study design using: randomized controlled clinical trial or pragmatic clinical trial with a comparator control group with another type of prenatal care. It is also recommended that data on socioeconomic and cultural aspects and satisfaction with prenatal care be collected. We similarly advise that both children and mothers be followed for a prolonged period after childbirth to check for other important outcomes related to the education received at the GPC.

## Data Availability Statement

The raw data supporting the conclusions of this article will be made available by the authors, without undue reservation.

## Ethics Statement

The studies involving human participants were reviewed and approved by the Research Ethics Committee of the University of Sorocaba, which is accredited by the National Research Ethics Commission according to stipulations of the National Health Council Ordinance 466/2012; it was also approved according to Platform Brazil Protocol no. 985.590 for March 13, 2015. It used only medical records, no identifiable data. Written informed consent from the participants' legal guardian/next of kin was not required to participate in this study in accordance with the national legislation and the institutional requirements.

## Author Contributions

LL and DH developed the original idea and the study protocol. DH, LL, IF, and MS performed the data collection, data analysis, and drafted the manuscript. All authors read and provided critical revision and approved the final manuscript.

## Conflict of Interest

The authors declare that the research was conducted in the absence of any commercial or financial relationships that could be construed as a potential conflict of interest.
